# The Relevance of Sex and Age as Non-Modifiable Risk Factors in Relation to Clinical-Pathological Parameters in Colorectal Cancer

**DOI:** 10.3390/life15020156

**Published:** 2025-01-23

**Authors:** Robert Barna, Alis Dema, Aura Jurescu, Adrian Ovidiu Văduva, Dorela-Codruța Lăzureanu, Octavia Vița, Bianca Natarâș, Ioana Hurmuz, Adelina Vidac, Sorina Tăban, Sorin Dema

**Affiliations:** 1 Department of Microscopic Morphology-Morphopatology, ANAPATMOL Research Center, “Victor Babes” University of Medicine and Pharmacy, 300041 Timisoara, Romania; 2 Department of Pathology, “Pius Brinzeu” County Clinical Emergency Hospital, 300723 Timisoara, Romania; 3 Department of Radiotherapy, Emergency City Clinical Hospital Timisoara, 300079 Timișoara, Romania; 4 Department of Oncology, “Victor Babeş” University of Medicine and Pharmacy, 300041 Timișoara, Romania

**Keywords:** colorectal cancer, age, sex, young patients, clinical-pathological parameters

## Abstract

Background and objectives: We aimed to assess the significance of sex and age compared to other clinical-pathological parameters in colorectal cancer (CRC). Materials and methods: Our study included a retrospective approach to CRC patients who underwent surgery at the ‘Pius Brinzeu’ County Clinical Emergency Hospital in Timisoara (PBECCHT), Romania. The analyzed parameters were: patient age and sex, tumor location, histological type, differentiation grade (G), extent of tumor (pT), lymph-node status (pN), distant metastasis status (pM), and lymphovascular invasion (LVI). The population was divided into three groups based on age, with those under 49 years old, 50 to 69 years old, and elderly (>70). Results: The study’s inclusion criteria were met by 1885 patients, with a male-to-female ratio of 1.39:1. There were significant differences between the sexes in the anatomical location of tumors (*p* < 0.0001). Younger patients were more likely to have deeply invasive tumors (*p* = 0.0096), LVI (*p* = 0.0332), lymph-node metastases (*p* = 0.0158), and metastatic disease (*p* = 0.0017). Conclusions: Over the ten-year period reviewed, the frequency of CRC cases has progressively increased, with males being diagnosed more often. In terms of patient age, the young population exhibits clinical features of aggressive evolution. Patient sex did not influence the analyzed parameters, except for tumor location, where right colon tumors are slightly more common in females.

## 1. Introduction

Globally, in 2022, colorectal cancer (CRC) ranked third in frequency, with 1,926,425 new cases, and second in mortality, with 904,019 deaths [1,2]. Over the past decades, a significant variation in CRC incidence has been observed worldwide, closely correlated with levels of socioeconomic development [3,4,5,6,7]. Incidence rates have peaked in many countries across Eastern Europe, Latin America, and Asia, where incidence had remained low for a long time [3,6,8,9].

Modifiable risk factors for CRC include physical inactivity [10], consumption of processed and red meat [11], alcohol consumption [12], and being overweight or obese [13,14,15,16]. Conversely, dietary fiber and dairy product consumption [17], physical activity [14], prolonged use of nonsteroidal anti-inflammatory drugs (NSAIDs), and hormone replacement therapy in females appear to reduce the risk of CRC [18].

Regarding non-modifiable risk factors, such as the sex and age of patients at diagnosis, incidence rates have been increasing in both males and females [19,20]. Overall, the incidence of CRC is higher in males (21.9 per 100,000) compared to females (15.2 per 100,000) [1,2,7]. The risk of CRC increases significantly after the age of 40, begins to rise sharply between ages 50–55, doubling with each decade, and continues to grow exponentially [21].

The trend of increasing CRC incidence in individuals younger than 50 years old (early-onset CRC patients—EOCRC) has been recently reported in Australia, Canada, and the United States (US) [7,9,22,23]. Some authors suggest that CRC incidence is rising among early-onset individuals, while it may be declining in older patients [2,23,24]. Moreover, EOCRC often presents at an advanced stage and is associated with adverse pathological characteristics that increase the risk of premature morbidity and death [9,23,25].

Currently, the molecular mechanisms underlying these epidemiological associations and risk factors for CRC are not fully understood [7], warranting further research in this area. Understanding the biological mechanisms regulated by sex and age could contribute to early diagnosis and favorable outcomes for patients with this neoplasm.

### Study Aim and Objectives

The aim of our study was to delineate the profile of patients with primary colorectal tumors by evaluating the relationship between non-modifiable risk factors (patient sex and age) and the key clinico-pathological characteristics of colorectal tumors: tumor location, histological type, tumor differentiation grade (G), depth of tumor invasion into the intestinal wall (pT), lymph node status (pN), distant metastasis status (pM), and lymphovascular invasion (LVI).

## 2. Materials and Methods

To achieve the stated objectives, we conducted a retrospective observational study and developed a database in an Excel table. The database included cases of primary CRC diagnosed through surgical resections performed on patients admitted to the Surgical Clinics of the PBECCHT, from January 2009 to December 2018.

### 2.1. Ethics Statement

The study was conducted in accordance with the Declaration of Helsinki and approved by the Ethics Committee of PBECCHT (Approval No. 459/15 April 2024). Informed consent was waived due to the retrospective nature of the study. However, at the time of hospitalization, patients provided consent under Romanian legislation by signing a form permitting the use of biological specimens for medical studies and the educational/scientific use of tissue or organ images.

### 2.2. Data Collection

Clinical data for the analyzed tumors were retrieved from medical records, while histopathological parameters were obtained from pathology reports in the Pathology Department database of PBECCHT.

#### 2.2.1. Inclusion Criteria

We established the following inclusion criteria:

● Consecutive cases of primary CRC diagnosed via histopathological examination of radical surgical resection specimens with regional lymphadenectomy.

#### 2.2.2. Exclusion Criteria

The exclusion criteria were:

● Patients with cancer types other than colorectal carcinomas.
● Patients with secondary colorectal tumors.
● Cases of CRC diagnosed via endoscopic biopsy or polypectomy specimens.
● Cases of recurrent CRC.
● Patients who underwent neoadjuvant radio-chemotherapy.

### 2.3. Histopathological Examination

Diagnoses of CRC were established through standard histopathological processing of surgical resection specimens in the PBECCHT Pathology Department. Tissue fragments were embedded in paraffin. Tissue sections 3–4 µm thick were cut from the paraffin blocks and stained using the routine hematoxylin-eosin (HE) method.

#### 2.3.1. Tumor Classification and Staging

The histological subtype and tumor differentiation grade were determined using WHO classifications [26,27] for digestive system tumors (editions used at the time of diagnosis).

Pathological staging was performed according to the pTNM system (AJCC/UICC editions applicable at the time of case evaluation) [28,29].

#### 2.3.2. Data Recorded

The following demographic and clinico-morphological parameters were extracted from histopathological reports and entered into the database:

● Patient age:
  • Subdivided into cohorts by decade (≤20 years, 21–30 years, 31–40 years… 91–100 years).
  • Grouped into three categories: Group I—patients under 49 years old, Group II—patients aged 50 to 69 years, and Group III—elderly (>70), according to the cutoffs used in other studies [30,31,32,33,34].
● Sex: Female (F) or male (M).
● Year of diagnosis.
● Tumor location:
  • Right colon: Tumors in the cecum, ascending colon, hepatic flexure, and transverse colon.
  • Left colon: Tumors in the splenic flexure, descending colon, sigmoid colon, and rectosigmoid junction.
  • Rectum.
● Histological type (WHO [27] criteria):
  • ADK NOS (classic, conventional type).
  • Mucinous adenocarcinoma: ≥50% extracellular mucinous secretion.
  • Adenocarcinoma with mucinous component: <50% mucinous component.
  • Signet-ring cell carcinoma: ≥50% signet-ring cells.
  • Adenocarcinoma with signet-ring cell component: <50% signet-ring cells.
  • Medullary carcinoma: Solid areas of poorly differentiated malignant cells with eosinophilic cytoplasm, vesicular nuclei, and prominent nucleoli.
● Tumor differentiation grade (G):
  • ADK NOS was graded according to the WHO [27] criteria, based on the percentage of gland formation:
    − G1: Well-differentiated (>95% gland formation).
    − G2: Moderately differentiated (50–95%).
    − G3: Poorly differentiated (0–49%).
    − G4: Undifferentiated (no gland formation, no mucin, or neuroendocrine or squamous differentiation).
  • Grouped into low-grade (G1–G2) and high-grade malignancy (G3–G4) [35].
● Depth of tumor invasion (pT):
  • Individual categories: pT1 (submucosa), pT2 (muscularis propria), pT3 (subserosal fat), pT4 (serosa).
  • Grouped: Early invasion (pT1–pT2) and deep invasion (pT3–pT4).
● Lymph node status (pN):
  • Individual categories: pN0, pN1, pN2.
  • Grouped: No lymph node metastases (pN0) or lymph nodal metastases present (pN1 + pN2).
● Distant metastases (pM1): Pathologically documented.
● Lymphovascular invasion (LVI): Presence/absence (LV1/LV0).
● Synchronous multiple tumors:
  • Defined as the presence of two or more distinct primary CRCs diagnosed within a six-month period. Tumors within the same intestinal segment were considered synchronous if they were at least 4 cm apart [36].
  • For statistical analysis of G and pT parameters, the characteristics of the main tumor (deepest invasion and most lymph nodes involved) were considered.

### 2.4. Statistical Analysis

Collected parameters were statistically analyzed using Microsoft Office Excel 2010 (Microsoft Corp., Redmond, WA, USA) and and GraphPad Prism software, v8.2 (GraphPad Software Inc., San Diego, CA, USA). The results were presented using counts and associated percentages for categorical data. Statistical differences in clinico-pathological parameters were evaluated using Chi-squared tests and Fisher’s exact test, with the corresponding *p*-values presented in the summary table. A *p*-value < 0.05 was considered statistically significant.

## 3. Results

Over the analyzed period (January 2009–December 2018), we identified 1885 cases of colorectal cancer (CRC) that met the inclusion criteria for the study. The patient cohort consisted of 1098 male patients (58.25%) and 787 female patients (41.75%), with a male-to-female ratio of 1.39:1. The patients’ ages ranged from 18 to 93 years, with an average age of 65.5 years. The average age for males was 65.6 years, and for females, it was 65.4 years.

The increasing trend in the number of cases during the analyzed period is observed in both males, rising from 75 cases (6.83%) in 2009 to 133 cases (12.11%) in 2018, and females, rising from 59 cases (7.50%) in 2009 to 96 cases (12.20%) in 2018, as shown in Table 1.

### 3.1. Evaluation of CRC Case Distribution by Age at Diagnosis and Patient Sex

From the distribution of CRC cases based on age at diagnosis, we observed that the tumor was predominantly diagnosed in Group II and III (patients over 50 years of age), accounting for 1730 cases—92% of the entire cohort. Regarding extreme age groups, a single case was identified in the under-20 age group (18 years old, male), while three cases were diagnosed in the over-90 age group, also male.

**Figure 1:**
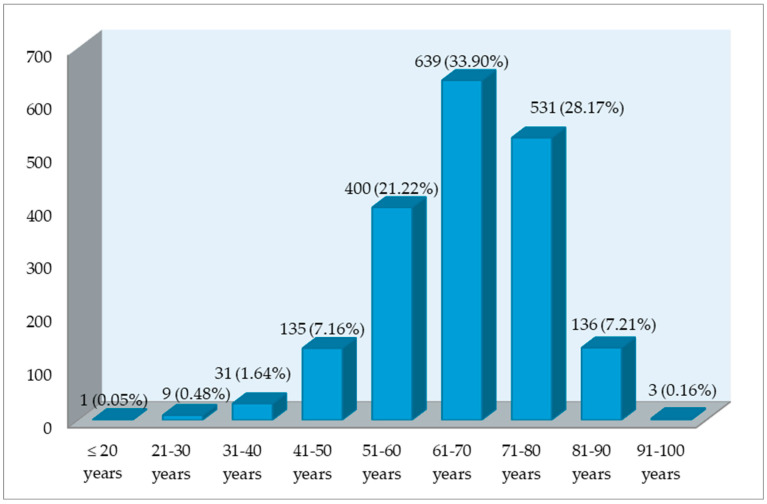
Distribution of CRC patients by age range at diagnosis at the ‘Pius Brinzeu’ County Clinical Emergency Hospital in Timisoara (PBECCHT), Romania (n = 1885).

The highest frequency of CRC cases was observed in the seventh decade of life (639 cases—33.90%), followed by the eighth decade (531 cases—28.17%), as shown in Figure 1.

The 61–70 age range represented the group with the highest frequency of CRC, both in males (n = 397; 36.16%) and females (n = 242; 30.75%). For females, the risk of CRC occurrence was nearly equal in the seventh (30.75%) and eighth decades of life (29.61%), Table 2.

In patients under 40 years, 24 cases (2.19%) were identified in males and 17 cases (2.16%) in females. In the 41–50 age range, the distribution of CRC cases was relatively balanced between sexes: 68 cases (6.19%) in males and 67 cases (8.51%) in females, as shown in Table 2.

### 3.2. Analysis of Synchronous Tumor Cases by Patient Age and Tumor Location

In 73 out of 1885 (4%) CRC cases, patients presented with synchronous tumors.

**Figure 2:**
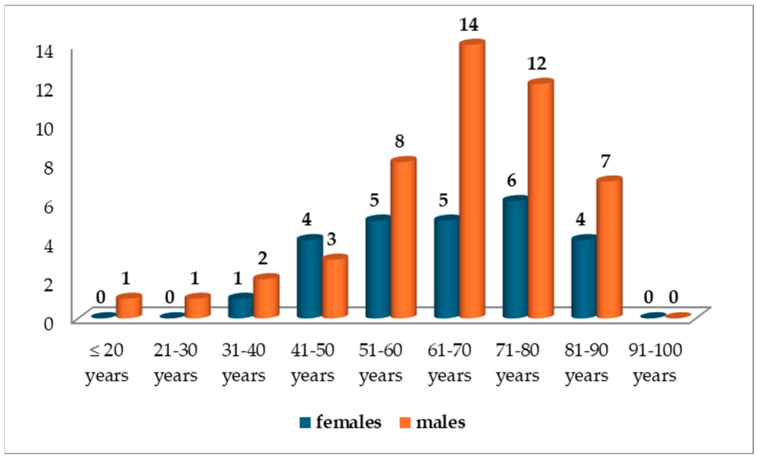
Distribution of cases with double synchronous tumors by patient sex and age decade at the ‘Pius Brinzeu’ County Clinical Emergency Hospital in Timisoara (PBECCHT), Romania (n = 73).

Synchronous tumors were diagnosed in patients aged between 18 and 90 years, with an average age at diagnosis of 64.8 years. The highest incidence of synchronous tumors occurred in the seventh and eighth decades of life: 19 cases (26.03%) and 18 cases (24.65%), respectively. In patients under 50 years of age, synchronous tumors were present in 12 out of 73 cases (16.44%), as shown in Figure 2.

In most cases (99%), double synchronous tumors were noted. Only one case—a male patient aged 18—was found to have multiple tumors: an adenocarcinoma NOS located at the hepatic flexure, two rectal adenocarcinomas NOS, and rectal polyps with high-grade dysplasia. This case was later confirmed to have familial colorectal polyposis.

Synchronous tumors were diagnosed more frequently in males, with 48 out of 73 cases (65.76%). In females, the most frequent synchronous tumor cases were found in the eighth decade of life (6 cases, 24%), while in males, they were more common in the seventh decade of life (14 cases, 29.17%), as shown in Figure 2.

The analysis of clinical-pathological parameters in cases of synchronous tumors represents a future research topic. In this study, for the evaluation of the primary tumor site and histological type, we did not consider the synchronous tumor cohort, as in most cases, we observed different locations along the intestinal tract as well as varying histological types. For the analysis of other pathological parameters in cases of synchronous tumors, we focused on the primary tumor, defined as the tumor with the deepest invasion into the intestinal wall.

### 3.3. Evaluation of Cases Based on Tumor Localization in the Large Intestine

From a topographical perspective, we analyzed the 1812 cases of CRC with a single tumor, after excluding the 73 cases of synchronous colorectal tumors from the original cohort of 1885 cases. We observed that the majority of cases (483 cases; 26.66%) were located in the rectum, followed by those in the sigmoid colon (439 cases; 24.23%) and the ascending colon (256 cases; 14.13%). The fewest cases were found at the rectosigmoid junction (138 cases; 7.62%) and at the cecum (151 cases; 8.33%).

In the transverse and descending colon, the incidence of CRC was nearly equal (171 cases—9.44% vs. 174 cases—9.60%). When grouping the cases into three categories, according to the classification established in the Section 2, we found that the most numerous tumors were in the left colon (41.45%—751 cases), followed by the right colon (31.90%—578 cases) and the rectum (26.66%—483 cases).

### 3.4. Evaluation of Cases Based on Histological Tumor Type

The distribution of the 1812 cases of CRC with a single tumor was as follows:

● Conventional adenocarcinomas (classic type, NOS)—1612 cases (88.96%)
● Mucinous adenocarcinomas—191 cases (10.54%)
● Signet-ring cell carcinomas—7 cases (0.39%)
● Medullary carcinomas—2 cases (0.11%).

Signet-ring cell carcinomas and medullary carcinomas were classified as “other types” for subsequent analyses.

### 3.5. Distribution of Cases Based on Histological Grade, Depth of Tumor Invasion (pT), Regional Lymph Node Status (pN), Presence of Distant Metastases (pM), and Lymphovascular Invasion (LVI)

From a histological grade perspective, 1578 (83.71%) cases were classified as low-grade carcinomas (G1–G2), while 307 cases (16.29%) were classified as high-grade carcinomas (G3–G4).

For the pT parameter, which reflects the depth of tumor invasion into the intestinal wall, we observed that only 39 tumors (2.07%) were classified as pT1, 219 cases (11.62%) as pT2, 1025 tumors (54.38%) surpassed the muscular layer and were classified as pT3, while 602 tumors (31.94%) had perforated the serosa and were classified as pT4.

When grouping the cases into two categories based on the depth of invasion (early invasion-pT1–pT2 vs. deep invasion into the intestinal wall-pT3–pT4), we found that the majority of cases—1627 tumors (86.31%)—belonged to the pT3-pT4 category.

Regarding regional lymph node status, we observed that in 946 cases (50.19%), no lymph node metastases were found (pN0). However, 517 cases (27.43%) were classified as pN1 and 422 cases (22.39%) as pN2. When grouping patients based on the absence or presence of lymph node metastases (pN0 vs. pN1+pN2), we noted that lymph node metastases were present in 939 cases (49.81%).

Distant metastases (pM1) were documented in 117 cases at the time of diagnosis. Lymphovascular invasion (LVI) was identified in 888 cases (47.11%).

### 3.6. Evaluation of the Relationship Between Patient Age and Other Clinicopathological Parameters

We further analyzed the relationship between clinicopathological parameters, considered as prognostic factors, such as tumor location, histological grade, tumor invasion into the intestinal wall, lymphovascular invasion (LVI), regional lymph node metastases, distant metastases, age, and sex of the patients, to observe their prognostic significance.

In terms of patient age, three subgroups were considered:

● Group I: patients ≤49 years old
● Group II: patients aged 50 to 69 years
● Group III: patients aged 70 to 93 years (see Table 3).

Patients in the age range of 50 to 69 years predominated (992 cases, 52.63%), followed by those over 70 years old (738 cases, 39.15%). From the distribution of cases by sex, we observed that tumors were more frequent in males across all three age groups.

Regarding the topography of cases with a single tumor, in Group I, tumors located in the left and right colon occurred in equal proportions (55 cases each, 38.19%), while in Groups II and III, tumors were more frequently diagnosed in the left colon (42.25% and 41.02% of cases, respectively). Rectal tumors were more commonly found in patients from Group II (284 cases, 29.55%), *p* = 0.003. Synchronous tumors were equally present in Groups II and III (31 cases, 42.47%).

In terms of histological type, non-mucinous adenocarcinoma (ADK) was identified in 130 cases (90.28%) in Group I, while slightly lower percentages were observed in the other two groups. Mucinous ADK was more frequently identified in Group III (86 cases, 12.16%).

Regarding the histological grade of tumors, G1–G2 tumors predominated in each age group, with no significant differences between age categories.

When evaluating the distribution of cases based on the pT parameter, we observed that tumors classified as pT1–pT2 were more frequent in Group II (150/992 cases, 15.13%), while pT3–pT4 tumors were more frequent in Group I (140/155 cases, 90.32%), *p* = 0.0096.

Concerning regional lymph node status, the most common cases of lymph node-negative metastasis (pN0) were found in Group II (395/738 cases, 53.52%). Lymph node metastases (pN1 + pN2) were more commonly encountered in younger patients—Group I (89/155 cases, 57.42%), *p* = 0.0158. Additionally, distant metastases (pM1) were most commonly found in Group I (11.61%, *p* = 0.0017). Lymphovascular invasion (LVI) was more frequently identified in Group I (87/155 cases, 56.13%), *p* = 0.0332 (see Table 3).

### 3.7. Analysis of the Relationship Between Sex and Clinicopathological Parameters

Regarding tumor location, females had a higher frequency of tumors located in both the left and right colon (593/787 cases—77.82%), while in males, tumors were more frequently located in the left colon and rectum (758/1098 cases—72.19%). Right colon tumors are slightly more common in female (37.53%) vs. in males (27.81%), with a statistically significant difference (*p* < 0.0001). Synchronous tumors were present in 25 cases (34.25%) in females and 48 cases (65.75%) in males.

Histological type:

● Non-mucinous adenocarcinoma (ADK) was identified in both males and females.
● Mucinous ADK was identified slightly more frequently in females (11.69% vs. 10.66%).

In terms of histological grade (G1–G2 vs. G3–G4), G1–G2 tumors predominated in both sexes, and similarly, pT3–pT4 tumors were more common in both sexes.

Regarding regional lymph node status (pN1, pN2), distant metastases (pM1), and lymphovascular invasion (LVI), the distribution of cases was relatively uniform between the sexes, as noted in Table 4.

## 4. Discussion

Colorectal cancer (CRC) represents a global challenge due to its increased incidence and the high morbidity and mortality rates in both sexes. This rise in incidence is believed to be the consequence of changes in dietary patterns, lack of physical activity, and the growing number of smokers [2,8,9,37]. In the United States, the incidence of CRC in males and females under 50 has steadily increased by 2% per year from 1995 to 2016 [4,38]. Some registries even report an increase in CRC incidence among young adults aged 20 to 39 years, although the absolute incidence in this age group remains much lower than in adults aged 50 and over [19,23].

In this study, we analyzed the demographic and clinicopathological characteristics of 1885 patients diagnosed with CRC over a 10-year period (January 2009–December 2018) at PBECCHT. We analyzed correlations between clinicopathological parameters (sex, age, tumor localization, histological type and grade, pT, pN, pM1, LVI) in an attempt to outline the profile of colorectal cancer patients and tumors in the western part of the country. In Romania, the incidence, prevalence, and mortality from CRC are on the rise [39]. According to national statistics, CRC incidence is higher in the western and southwestern regions of the country, possibly due to unhealthy dietary habits, particularly the high consumption of red and processed meats [40]. In our study, we observed that the number of patients undergoing radical CRC surgery progressively increased over the analyzed 10-year period, with a 71% increase in the number of cases diagnosed in 2018 compared to 2009. However, this study does not reflect the actual CRC incidence in the western part of the country, as there are other hospitals/medical centers where patients are diagnosed and operated for CRC. Additionally, we included only cases of CRC diagnosed from surgical resection specimens, excluding those diagnosed from preoperative biopsies or patients who had received chemotherapy/radiotherapy prior to surgery.

Regarding the presence of synchronous colorectal tumors, our results show the occurrence of double synchronous tumors in 73 cases (4%), an incidence very close to that reported by other studies—3.5% (with variability ranging from 3.1% to 3.9%) of all colorectal cancers [36,41,42]. Synchronous CRC is more commonly reported in males, with a male-to-female ratio of 1.8:1, in the first half of the seventh decade of life [36]. In this regard, our findings, which show that the average age at diagnosis for patients with synchronous CRC was 64.8 years and that synchronous tumors were more frequently diagnosed in males (65.76%), align with data from other studies [36]. It is known that patients with inflammatory bowel diseases (ulcerative colitis and Crohn’s disease), HNPCC, FAP, and serrated polyps/adenomas have a higher risk of synchronous CRC [41], but we did not analyze such associations/conditions. Additionally, the literature describes that most patients with synchronous CRC have two carcinomas, although up to six tumors have been reported in a single patient [36]. Unlike the literature on this topic, we predominantly identified double synchronous tumors. Although the literature suggests that synchronous tumors are more frequently found in the right colon compared to solitary CRC [36], in our study, the majority of synchronous tumors were diagnosed in the left colon. In general, the prognosis for patients with synchronous CRC is not significantly different from that of patients with solitary CRC [42]. A different approach to therapy and long-term clinical surveillance is recommended for some patients with synchronous tumors. Therefore, identifying patients at high risk for synchronous CRC, as well as understanding prognostic factors, is essential as complementary tools for recommending personalized therapy.

Age is considered a major risk factor for sporadic colorectal cancer (CRC) [9,33]. CRC is less commonly encountered before the age of 40, but its incidence starts to rise significantly between the ages of 40 and 50, with incidence rates doubling every subsequent decade [9]. In the United States, patients over 65 years old are three times more likely to be diagnosed with CRC than those aged 50–64, and approximately 30 times more likely than those aged 25–49 [7]. The literature mentions that around 90% of all CRC cases are diagnosed after the age of 50, with the increase in age directly correlating, even exponentially, with the risk of CRC [21]. Regarding the age at diagnosis of CRC patients, our data align with the literature, as 1709/1885 (90.67%) patients were over 50 years old, with the average age of the entire cohort being 65.5 years. Multivariate analysis of parameters based on the three age categories showed statistically significant correlations with tumor location (*p* = 0.030): in patients under 50 years of age and over 70 years of age, most tumors were located in the right and left colon. In patients aged 50–69 years, there was a higher frequency of cases in the rectum compared to other age categories. In Group I, there were more frequent tumors with deep extension into the wall (90.32%, *p* = 0.0096), lymph node metastases (57.42%, *p* = 0.0158). Additionally, LVI was more frequently identified in cases from Group I (56.13%, *p* = 0.0332), and distant metastases were found in 11.61% (*p* = 0.0017). Our results support the data from the literature, which suggests that younger patients with CRC have a poor prognosis due to lesions being most often detected at a late stage, which is more advanced [19,23].

Approximately 3% of young patients under the age of 30 may develop colorectal cancer (CRC), but in 11% of these cases, familial adenomatous polyposis (FAP) or ulcerative colitis (UC) are identified in the medical history [15]. In these young patients, tumors are typically detected late, usually in advanced stages, are generally poorly differentiated, and mucinous and “signet ring” carcinoma subtypes predominate, which is why the prognosis is more reserved [43]. Morbidity due to CRC increases significantly with age. The incidence and mortality of CRC in patients over 70 years old are higher in females than in males [44]. Additionally, the 5-year survival rate for CRC is lower in females than in men, with significant differences in females over 70 years old [7]. In patients over 70 years of age, the prognosis is influenced by the success of complete surgical excision and is more reserved due to associated pathologies and/or advanced disease stage, as well as higher postoperative mortality. Modern treatment protocols have improved the prognosis of these patients [43,45].

Regarding sex, the male-to-female ratio was 1.39:1, which is consistent with other observations in the literature that report CRC being diagnosed more frequently in men, who also have a mortality rate from CRC approximately 25% higher than females [46]. In the case of conventional adenocarcinoma (ADK), the incidence was also higher in males. We observed significant differences between sexes regarding the anatomical location of tumors (*p* < 0.0001). In women, tumors were more frequently located in the right colon (37.53%), compared to males (27.81%). Our results confirm/reinforce findings from similar studies that indicate an association between tumor location and sex, with CRC being more common in the proximal colon in females [47]. Hansen et al. reported that a higher proportion of females present with proximal colon cancer, and it is often diagnosed at a more advanced stage [47]. Therefore, the lower 5-year survival rate in females over 70 years old may be related to this phenomenon [44]. Another study, which analyzed how tumor primary location and age at diagnosis affect the risk of CRC recurrence [48], showed that this risk increased both in patients with proximal colon cancer and those with distal colon cancer in the first 5 years after the initial diagnosis, even among young patients (*p* < 0.0001) [48]. The study also found that patients with recurrent cancer, who initially presented with a tumor in the proximal colon, were more likely to be elderly, female, and have poorly differentiated tumors compared to those with distal colon or rectal cancer. The overall risk of recurrence increased among patients with cancer initially located in the right and left colon, but not in those with rectal cancer [48].

Colorectal cancer (CRC) is one of the most common causes of morbidity in both males and women, exhibiting different molecular and pathological characteristics depending on the tumor’s location. Generally, CRC is diagnosed more frequently in men, and the mortality rate from CRC is approximately 25% higher in males than in females [46]. However, females over the age of 65 have a higher mortality rate from CRC and a lower 5-year survival rate compared to males [49]. Females are at a higher risk of developing right-sided colon cancer, which is associated with a greater frequency of neoplasm in more advanced stages and poorer outcomes [47]. CRC is a major threat to the health of older women, and the lower 5-year survival rate in females may be due to the increased incidence of proximal colon cancer, which, due to vague symptoms, is diagnosed at a more advanced stage and is associated with a more aggressive form of neoplasm compared to distal colon cancer [49,50].

Some hormonal factors may explain the higher percentage of proximal CRC in women. According to Schoenfeld et al., CRC appears to have a predilection for the proximal colon in females [51], especially in those who are postmenopausal. Another study reported that the lack of estrogen in older women increased the risk of MSI-H (microsatellite instability-high) colon cancer [52]. Furthermore, postmenopausal females using hormone replacement therapy (HRT) exhibited a 40% reduction in CRC risk, while females using HRT after being diagnosed with CRC showed a more advanced stage of CRC [53]. These results suggest that hormone replacement therapy could have a detrimental effect on CRC once the tumor has developed. Overall, according to some studies, HRT is initially associated with protection against CRC [33], but once the tumor has developed, estrogens may promote CRC proliferation, especially in postmenopausal females [49,53,54].

The impact of tumor location on survival is still unclear, so more research is required to investigate pathological differences related to tumor location and patient sex to develop appropriate CRC screening and treatment strategies.

## 5. Study Limitations

This study has several limitations, some of which have been mentioned in previous paragraphs. Additionally, the most significant limitation is the absence of data regarding the treatment received by the patients or their post-surgery progress, which would allow for correlating the results obtained with survival rates and significantly improving our work. However, the total number of CRC patients included in the study is relevant for the study’s objectives. Unfortunately, national oncology registers are not currently available in our country. Patients can present at regional hospitals, but there is no uniform reporting of cases and cancer types, which prevented us from tracking the patients or accurately assessing the incidence, prevalence, and mortality related to the disease. Another limitation is the lack of information regarding the patients’ family cancer history and the presence of other risk factors. Moreover, our study is limited by the fact that the analysis was conducted retrospectively and evaluated patients diagnosed at a single center.

Therefore, by analyzing data from cancer registries, it would be possible to assess how biological factors such as sex and age influence the clinical and biological characteristics of tumors. A complete understanding of these factors would allow the development of appropriate screening and prevention strategies, which would contribute to the early diagnosis of CRC and the establishment of specific therapeutic protocols adapted according to the patients’ age and sex, aimed at improving the quality of life and survival of patients.

## 6. Conclusions

The frequency of colorectal carcinomas cases has progressively increased over the analyzed ten-year period, with most cases being diagnosed in males. Regarding patient age, the seventh decade represented the age group with the highest incidence of carcinomas in both sexes. In younger patients (under 50 years old), deeply invasive tumors, lympho-vascular invasion, lymph-node metastases, and metastatic disease were more frequent. As for patient sex, no statistically significant differences were observed between the analyzed parameters, except for tumor location, right colon tumors being slightly more frequent in females. Additionally, synchronous double tumors were predominantly located in the left colon and were mainly diagnosed in male patients.

## Figures and Tables

**Table 1:** Annual distribution and sex-based distribution of CRC cases at the ‘Pius Brinzeu’ County Clinical Emergency Hospital in Timisoara (PBECCHT), Romania (n = 1885).

Year of Diagnosis	No of Cases	Annual Percent Change	Males	Annual Percent Change	Females	Annual Percent Change
year 2009	134 (7.11%)		75 (6.83%)		59 (7.50%)	
year 2010	155 (8.22%)	15.67	90 (8.20%)	20	65 (8.26%)	10.17
year 2011	171 (9.07%)	10.32	99 (9.02%)	10	72 (9.15%)	10.77
year 2012	197 (10.45%)	15.20	115 (10.47%)	16.16	82 (10.42%)	13.89
year 2013	199 (10.56%)	1.02	115 (10.47%)	0	84 (10.67%)	2.44
year 2014	202 (10.72%)	1.51	125 (11.38%)	8.70	77 (9.78%)	−8.33
year 2015	194 (10.29%)	−3.96	113 (10.29%)	−9.60	81 (10.29%)	5.19
year 2016	191 (10.13%)	−1.55	112 (10.20%)	−0.88	79 (10.04%)	−2.47
year 2017	213 (11.30%)	11.52	121 (11.02%)	8.04	92 (11.69%)	16.46
year 2018	229 (12.15%)	7.51	133 (12.11%)	9.92	96 (12.20%)	4.35

**Table 2:** Distribution of CRC cases by patient sex and age categories at the ‘Pius Brinzeu’ County Clinical Emergency Hospital in Timisoara (PBECCHT), Romania (n = 1885).

Patients	≤20 Years	21–30 Years	31–40 Years	41–50 Years	51–60 Years	61–70 Years	71–80 Years	81–90 Years	91–100 Years
Males (%)	1 (0.09)	6 (0.55)	17 (1.55)	68 (6.19)	230 (20.95)	397 (36.16)	298 (27.14)	78 (7.10)	3 (0.27)
Females (%)	0	3 (0.38)	14 (1.78)	67 (8.51)	170 (21.60)	242 (30.75)	233 (29.61)	58 (7.37)	0

**Table 3:** The analysis of the relationship between clinicopathological parameters and patient’s age.

Parameters	Group I 18–49 Years		Group II 50–69 Years		Group III 70–93 Years		Chi Squared/ Fisher’s Exact Test
No cases	155	(%)	992	(%)	738	(%)	*p* value
Females	70	45.16	407	41.03	310	42.01	0.6145
Males	85	54.84	585	58.97	428	57.99	
Synchronous tumors	11	15.07	31	42.47	31	42.47	
Right colon	55	38.19	271	28.20	252	35.64	0.0030
Left colon	55	38.19	406	42.25	290	41.02	
Rectum	34	23.61	284	29.55	165	23.34	
Nonmucinous ADK	130	90.28	864	89.91	618	87.41	0.4859
Mucinous ADK	13	9.03	92	9.57	86	12.16	
Other types	1	0.69	5	0.52	3	0.42	
G1–G2	128	82.58	837	84.38	613	83.06	0.7069
G3–G4	27	17.42	155	15.63	125	16.94	
pT1	0	0.00	31	3.13	8	1.08	0.0096
pT2	15	9.68	119	12.00	85	11.52	
pT3	78	50.32	535	53.93	418	55.83	
pT4	62	40.00	307	30.95	227	31.57	
pN0	66	42.58	485	48.89	395	53.52	0.0158
pN1	44	28.39	269	27.12	204	27.64	
pN2	45	29.03	238	23.99	139	18.83	
Mx	137	88.39	925	93.25	706	95.66	0.0017
pM1	18	11.61	67	6.75	32	4.34	
LV0	68	43.87	521	52.52	408	55.28	0.0332
LV1	87	56.13	471	47.48	330	44.72	

ADK—adenocarcinoma. G—tumor differentiation grade, grading based on the percentage of gland formation: G1 (well-differentiated); G2 (moderately differentiated; G3 (poorly differentiated); G4 (undifferentiated). Cases are grouped into low-grade (G1–G2) and high-grade malignancy (G3–G4). pT—depth of tumor invasion: pT1 (submucosa), pT2 (muscularis propria), pT3 (subserosal fat), pT4 (serosa). pN—lymph node status: pN0 (no lymph node metastases), pN1 (metastasis in 1–3 regional lymph nodes), pN2 (metastasis in 4 or more regional lymph nodes). Mx absence/presence of distant metastases is unknown, pM1—pathologically documented distant metastases. LV—lymphovascular invasion: LV0 (absence), LV1 (presence).

**Table 4:** Analysis of the relationship between clinicopathological parameters and patient sex.

	Females		Males		Chi Squared/ Fisher’s Exact Test
No cases	787	%	1098	%	
Group I (18–49 years)	70	8.89	85	7.74	0.6145
Group II (50–69 years)	407	51.72	585	53.28	
Group III (70–93 years)	310	39.39	428	38.98	
Synchronous tumors	25	34.25	48	65.75	
Right colon	286	37.53	292	27.81	<0.0001
Left colon	307	40.29	444	42.29	
Rectum	169	22.18	314	29.90	
Nonmucinous ADK	692	87.93	974	88.71	0.5941
Mucinous ADK	92	11.69	117	10.66	
Other types	3	0.38	7	0.64	
G1–G2	661	83.99	917	83.52	0.7833
G3–G4	126	16.01	181	16.48	
pT1	13	1.65	26	2.37	0.3283
pT2	102	12.96	117	10.66	
pT3	426	54.13	599	54.55	
pT4	246	31.26	356	32.42	
pN0	396	50.32	550	50.09	0.8464
pN1	211	26.81	306	27.87	
pN2	180	22.87	242	22.04	
Mx	734	93.27	1034	94.17	0.4216
pM1	53	6.73	64	5.83	
LV0	415	52.73	582	53.01	0.9066
LV1	372	47.27	516	46.99	

ADK—adenocarcinoma. G—tumor differentiation grade, grading based on the percentage of gland formation: G1 (well-differentiated); G2 (moderately differentiated; G3 (poorly differentiated); G4 (undifferentiated). Cases are grouped into low-grade (G1–G2) and high-grade malignancy (G3–G4). pT—depth of tumor invasion: pT1 (submucosa), pT2 (muscularis propria), pT3 (subserosal fat), pT4 (serosa). pN—lymph node status (pN): pN0 (no lymph node metastases), pN1 (metastasis in 1–3 regional lymph nodes), pN2 (metastasis in 4 or more regional lymph nodes). Mx absence/presence of distant metastases is unknown, pM1—pathologically documented distant metastases. LV—lymphovascular invasion: LV0 (absence), LV1 (presence).

## Data Availability

All data generated or analyzed during this study are included in this published article and can be provided if needed or requested by the reviewer.
